# Increased syphilis testing and detection of late latent syphilis among women after switching from risk-based to opt-out testing strategy in an urban Australian sexual health clinic: a retrospective observational study

**DOI:** 10.1016/j.lanwpc.2023.100875

**Published:** 2023-08-07

**Authors:** Palak Gupta, Christopher K. Fairley, Marcus Y. Chen, Catriona S. Bradshaw, Glenda Fehler, Erica L. Plummer, Lenka A. Vodstrcil, Julien Tran, Ei T. Aung, Eric P.F. Chow

**Affiliations:** a Melbourne Sexual Health Centre, Alfred Health, Melbourne, Victoria, Australia; b Central Clinical School, Faculty of Medicine, Nursing and Health Sciences, Monash University, Melbourne, Victoria, Australia; c Melbourne School of Population and Global Health, The University of Melbourne, Melbourne, Victoria, Australia

**Keywords:** Syphilis, Women's health, Sexually transmitted infection, Sexually transmitted disease, Screening

## Abstract

**Background:**

The Melbourne Sexual Health Centre (MSHC) implemented an opt-out syphilis test for women in December 2017. We aimed to examine the differences in syphilis testing uptake and confirmed syphilis cases among women after switching from risk-based to opt-out testing strategies.

**Methods:**

This was a retrospective study examining all women attending the MSHC for the first time in periods of risk-based testing (2015–2017) and opt-out testing (2018–2020). We calculated the proportion of women who tested for syphilis and the proportion of women with confirmed syphilis in each period. A chi-square test was performed to determine the differences in proportion between the risk-based testing and opt-out periods.

**Findings:**

A total of 27,481 women (i.e. 13,059 in the risk-based testing period and 14,422 in the opt-out period) were included in the final analysis, and the mean age was 26.8 years (standard deviation = 6.9). The proportion of women who were tested for syphilis at their first consultation increased from 52.8% (6890/13,059) in the risk-based testing period to 67.4% (9725/14,422) in the opt-out period (p < 0.0001). Syphilis positivity did not differ between the two periods (0.48% [33/6890] vs 0.71% [69/9725], p = 0.061) but late latent causes increased from 36.4% [12/33] to 60.9% [42/69] (p = 0.033).

**Interpretation:**

The opt-out testing strategy increased syphilis testing among women with increased detection of asymptomatic late latent syphilis. The opt-out syphilis testing strategy is beneficial in sexual health services. Health education and awareness may be required to improve syphilis testing uptake.

**Funding:**

10.13039/501100000925National Health and Medical Research Council.

**Research in context**

**Evidence before this study**

There has been an increase in syphilis among women and a re-emergence of congenital syphilis in Australia since the late 2010s. Many countries (including Australia) do not recommend asymptomatic syphilis screening among women. We searched PubMed on May 22, 2023, using the terms (“syphilis” OR “*Treponema pallidum*”) AND (“women” OR “females”) AND (“screening” OR “testing”) AND “Australia”. We identified 219 studies using these search terms. The majority of studies focused on the prevalence, trend, epidemiology and risk factors for syphilis among women. We did not identify any studies examining the impact of using an opt-out syphilis screening approach in women.

**Added value of this study**

To our best knowledge, this is the first study to examine the impact and significance of using an opt-out syphilis screening approach in women. We performed a retrospective observational study by including >27,000 women from a large Australian urban sexual health clinic in 2015–2020. By comparing with the risk-based syphilis testing approach (2015–2017), we found that the opt-out syphilis testing approach (2018–2020) could increase syphilis screening in women and also lead to an increase in the detection of asymptomatic late latent syphilis.

**Implications of all the available evidence**

Upward pressure on syphilis incidence in women is likely to continue not only in Australia but also in other settings. There have been a few studies examining the benefits of an opt-out syphilis testing approach but these studies are primarily conducted among people living with HIV or men who have sex with men. Our study provides evidence demonstrating the benefits of an opt-out syphilis testing approach in women. This is one of the significant approaches to preventing congenital syphilis as well as more serious complications of syphilis such as neurosyphilis and ocular syphilis.

## Introduction

The infectious syphilis notification rate has increased dramatically in Australia over the last 10 years, from 5 per 100,000 population in 2010 to 23.9 per 100,000 population in 2020.^1^ A sharp increase in syphilis cases among heterosexual men and women, especially those of child-bearing age, Aboriginal and Torres Strait Islander peoples and women pregnant at the time of diagnosis, has caused considerable concern.^2^, ^3^, ^4^, ^5^ In particular, the increasing rate of syphilis in women of reproductive age has been identified as a public health priority due to its association with congenital syphilis, which is complicated by congenital defects, prematurity, low birth weight and stillbirth.^1^^,^^6^^,^^7^ In Victoria, the proportion of syphilis cases that are women increased from 8.8% in 2016 to 12.8% in 2020.^8^^,^^9^ In Victoria, there were no congenital syphilis cases since the last single case in 2004; however, there has been a re-emergence of congenital syphilis cases since 2017 (14 cases in 2017–2022).^10^

Current Australian sexual health and general practice guidelines^11^, ^12^, ^13^, ^14^, ^15^ do not recommend screening asymptomatic heterosexual women for syphilis unless they engage in sex with men who have sex with men (MSM).^11^ The Syphilis Communicable Disease Network Australia (CDNA) National Guidelines for Public Health Units recommends syphilis testing when there is a clinical presentation suspicious of syphilis, diagnosis of another sexually transmitted infection (STI), contact of infection or upon request.^11^ The CDNA National Guidelines,^11^ Pregnancy Care Guidelines^12^ and The Royal Australian College of General Practitioners (RACGP)^13^ advise routine syphilis testing for pregnant women at first antenatal contact and again at 28 weeks for high-risk women.

Timely testing, diagnosis and treatment are important to prevent and control syphilis. Several settings have demonstrated that implementing an opt-out syphilis testing strategy (i.e. syphilis testing performed automatically when blood is collected as part of routine HIV monitoring) could increase the annual syphilis testing frequency.^16^^,^^17^ This practice has been shown to be associated with an increase in the detection of early latent syphilis along with a relative reduction in secondary syphilis.^16^^,^^17^ However, most of the previous studies were conducted among at-risk populations such as MSM and people living with HIV (PLHIV), and there have been limited studies evaluating the opt-out syphilis testing strategy in women.

The Melbourne Sexual Health Centre (MSHC) is the major public HIV/STI clinic located in Melbourne, Australia, and the clinic changed the syphilis testing strategy for women from risk-based to an opt-out testing strategy in December 2017. This study aimed to examine the changes in the proportion of women who tested for syphilis, the number and proportion of women with confirmed syphilis between the risk-based testing and opt-out periods.

## Methods

### Study setting

The study was conducted at the MSHC, which is the largest provider of sexually transmitted infection (STI) services in Victoria. MSHC provides primarily free HIV/STI testing and treatment to all individuals, at risk of STIs and is not a primary provider of reproductive health services. The clinic provides approximately 50,000 consultations a year and about one-third of the consultations are women. As part of routine procedures, clients are asked to complete a demographic and sexual practices questionnaire via computer-assisted self-interviewing (CASI) before being seen by a health care practitioner.

### Changes in syphilis testing strategy

In December 2017, MSHC changed the syphilis testing strategy for women to respond to and address the rise in syphilis among women in Victoria. Before December 2017, the clinic employed a risk-based syphilis testing strategy based on the standard of care. Syphilis testing was primarily performed for people presenting with related symptoms or reported sexual contact with partners with an STI. From December 2017 onwards, an opt-out syphilis testing strategy was implemented. Clinicians were encouraged to recommended a syphilis test to all clients regardless of their risk and demographic characteristics, and clients were able to decline testing if they did not want it.

### Study population

Electronic data from January 2015 to December 2020 were extracted. We included individuals if they were (1) cis female; (2) aged 16 years or above; (3) not engaged in sex work; and (4) had attended MSHC for the first time during the study period. We only included first consultations because testing at previous visits would have influenced testing at future consultations.^18^ We excluded female sex workers because sex workers in Victoria were required to have a mandatory 3-monthly HIV/STI (including syphilis) testing in accordance with the sex work regulations.^19^ In this analysis, we categorised the study period into (i) a risk-based testing period (2015–2017) and (ii) an opt-out testing period (2018–2020) with each period containing three calendar years.

The primary outcomes were the proportion of women who were tested for syphilis, the number and proportion of women with confirmed syphilis in each period. Testing for syphilis involves screening with chemiluminescence immunoassay (CLIA) (Diasorin, Saluggia, Italy) and a positive CLIA test was confirmed by rapid plasma reagin (RRP) test, *T. pallidum* particle agglutination (TPPA) assay, and a recombinant total antibody ELISA immunoassay (EIA). Sexual health clinicians reviewed all clients with positive syphilis serology results and categorised the syphilis cases that require treatment into four clinical stages of syphilis (primary, secondary, early latent and late latent) as per the Australian guidelines. If there is too little information to clarify cases into one of those categories, they are categorised as “unknown”. If an individual is tested positive but does not require treatment, they are categorised as ‘past treated syphilis’ and not included as cases in our analysis. A confirmed syphilis case was defined as having both a positive result on the confirmatory test and requiring treatment by clinician evaluation of history.

### Statistical analyses

A chi-square test was used to determine the differences in the proportion of women who were tested for syphilis and proportion of women with confirmed syphilis between the risk-based testing and opt-out periods. Fisher's exact test was used to determine the differences in the proportion of syphilis cases that were primary, secondary, early latent and late latent between the two periods. We also calculated the annual proportion of syphilis and a chi-square trend test was used to determine whether there was a change during the study period. We calculated the proportion of women who were tested for urogenital chlamydia during the study period as a proxy for sexual risk. A separate category “unknown/missing” was created for independent variables with missing or unknown values. We performed additional analysis to calculate the proportion of women who were tested for syphilis in the opt-out testing period by their demographic characterises and sexual practices; hence, women with low syphilis testing in certain groups can be encouraged to test using the opt-out testing strategy in clinical practice. All analyses were performed in Stata (version 17, College Station, TX, USA). This study was approved by the Alfred Hospital Ethics Committee, Melbourne, Australia (Project Number 123/21).

### Role of funding source

EPFC is supported by an Australian National Health and Medical Research Council (NHMRC) Emerging Leadership Investigator Grant (GNT1172873). CKF and CSB are supported by an Australian NHMRC Leadership Investigator Grant (GNT1172900 for CKF and GNT1173361 for CSB). ETA is supported by the Australian Government Research Training Program (RTP) scholarship from Monash University and the Research Entry Scholarship from the Chapter of Sexual Health Medicine, Royal Australasian College of Physicians. JT is supported by the Australian Government Research Training Program (RTP) Scholarship from Monash University. The funders of the study had no role in the design or conduct of the study, including data collection, management, analysis, or interpretation of the results; preparation, review, or approval of the manuscript; or the decision to submit the manuscript for publication.

## Results

**Fig. 1:**
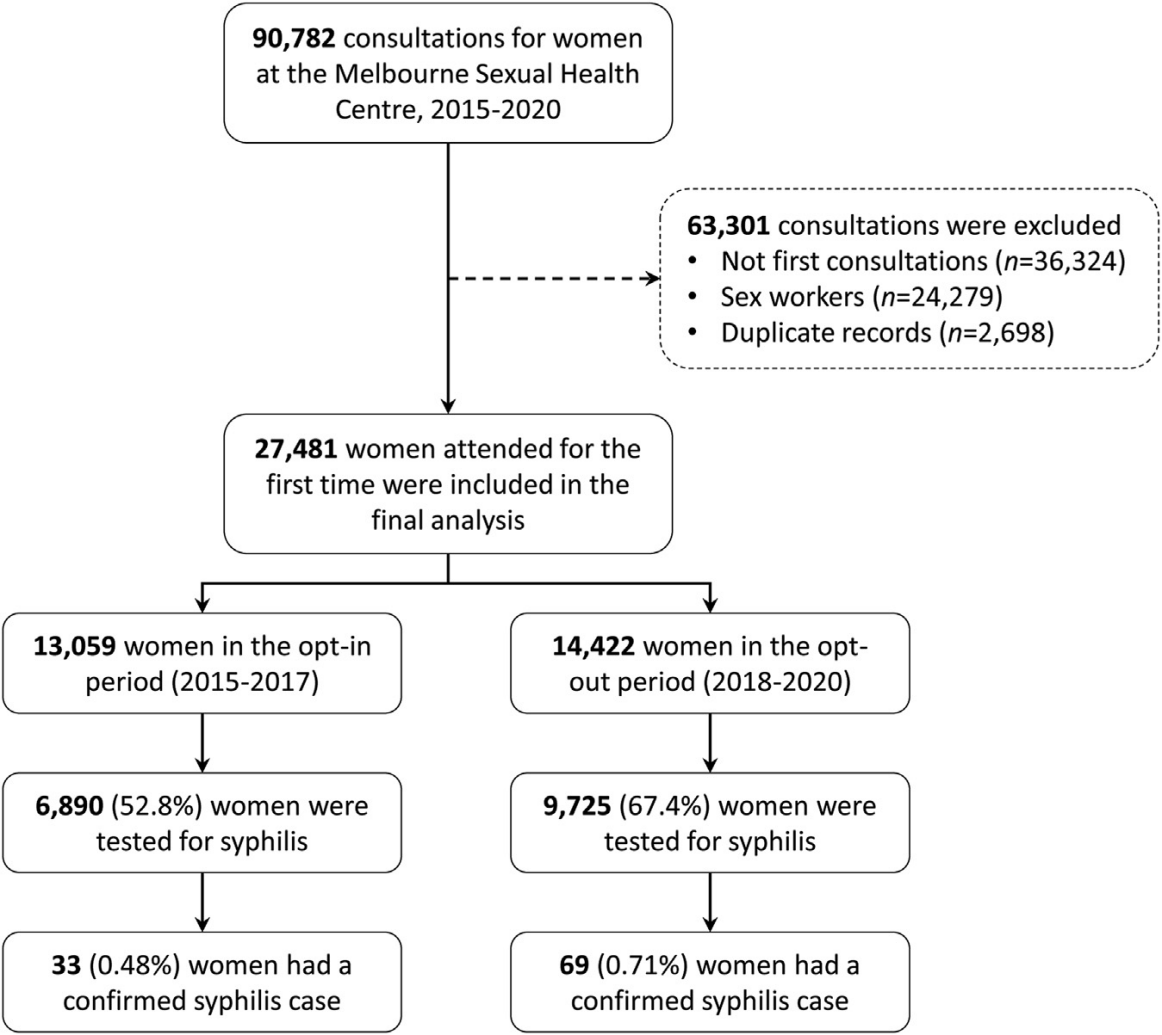
**Flow chart of the selection criteria for final analysis**.

There were 90,782 consultations for women at MSHC between January 2015 and December 2020. We excluded 63,301 consultations because they were not first consultations (*n* = 36,324), sex workers (*n* = 24,279) and duplicate records (*n* = 2698) (Fig. 1). The remaining 27,481 consultations (i.e. 13,059 in the risk-based testing period and 14,422 in the opt-out period) fulfilled the inclusion criteria and were included in the final analyses.

The mean age of the 27,481 women was 26.8 years (standard deviation = 6.9) (Table 1). A small proportion of women were identified as Aboriginal and/or Torres Strait Islander (1.3%, 357/27,481) and more women were born outside Australia (65.2%, 17,092/27,481). Some demographic characterises were statistically significant between the two periods; however, the changes in proportions between the two periods were minimal.

**Table 1:** Demographic features of the participants between the risk-based testing and opt-out period.

	Risk-based testing (N = 13,059)	Opt-Out (N = 14,422)	Overall (N = 27,481)	p value^a^
Age, mean (SD)	26.70 (7.0)	26.9 (6.7)	26.8 (6.9)	0.006
Aboriginal and Torres Strait Islander				
Yes	186 (1.4%)	171 (1.2%)	357 (1.3%)	<0.0001
No	11,301 (86.5%)	11,642 (80.7%)	22,942 (83.5%)	
Declined to answer	1572 (12.0%)	2610 (18.1%)	4182 (15.2%)	
Country of birth				
Australia	3727 (28.5%)	3843 (26.6%)	7570 (27.6%)	<0.0001
Overseas	8602 (65.9%)	9301 (64.5%)	17,903 (65.1%)	
Declined to answer	730 (5.6%)	1278 (8.9%)	2008 (7.3%)	
People living with HIV				
Yes	17 (0.1%)	31 (0.2%)	48 (0.2%)	0.093
No	13,042 (99.9%)	14,391 (99.8%)	27,433 (99.8%)	
Reported ever injecting drugs				<0.0001
Never injected	12,237 (93.7%)	13,314 (92.3%)	25,551 (93.0%)	
Ever injected	117 (0.9%)	153 (1.1%)	270 (1.0%)	
Unknown/missing	705 (5.4%)	955 (6.6%)	1660 (6.0%)	
Pregnancy status				<0.0001
Not Pregnant	59 (0.5%)	90 (0.6%)	149 (0.5%)	
Pregnant	11,088 (84.9%)	12,223 (84.8%)	23,331 (84.9%)	
Unsure	987 (7.6%)	945 (6.6%)	1932 (7.0%)	
Unknown/missing	925 (7.1%)	1164 (8.1%)	2089 (7.6%)	
Gender of sexual partners in the last 12 months				<0.0001
Male only	11,039 (84.5%)	11,879 (82.4%)	22,918 (83.4%)	
Female only	141 (1.1%)	198 (1.4%)	339 (1.2%)	
Both male and female	898 (6.9%)	1112 (7.7%)	2010 (7.3%)	
Unknown/missing	981 (7.5%)	1233 (8.5%)	2214 (8.1%)	
Condomless sex with any male partner in the last 12 months				0.001
No	1446 (11.1%)	1605 (11.1%)	3051 (11.1%)	
Yes	10,289 (78.8%)	11,155 (77.3%)	21,444 (78.0%)	
Unknown/missing	1324 (10.1%)	1662 (11.5%)	2986 (10.9%)	
Number of male partners in the last 12 months, mean (SD)	2.3 (0.6)	2.3 (0.7)	2.3 (0.7)	0.163
Had regular male partners				<0.0001
No	6438 (49.3%)	6986 (48.4%)	13,424 (48.8%)	
Yes	5056 (38.7%)	5505 (38.2%)	10,561 (38.4%)	
Unknown/missing	1565 (12.0%)	1931 (13.4%)	3496 (12.7%)	
Had casual male partners in the last 12 months				0.009
No	1586 (12.1%)	1888 (13.1%)	3474 (12.6%)	
Yes	10,335 (79.1%)	11,194 (77.6%)	21,529 (78.3%)	
Unknown/missing	1138 (8.7%)	1340 (9.3%)	2478 (9.0%)	
Sex overseas in the last 12 months				<0.0001
No	6154 (47.1%)	6791 (47.1%)	12,945 (47.1%)	
Yes	5856 (44.8%)	6246 (43.3%)	12,102 (44.0%)	
Unknown/missing	1049 (8.0%)	1385 (9.6%)	2434 (8.9%)	
Tested for syphilis				
Yes	6890 (52.8%)	9725 (67.4%)	16,615 (60.5%)	<0.0001
No	6169 (47.2%)	4697 (32.6%)	10,866 (39.5%)	

a Two-sample t-test was used to compare the mean age between the two periods. Chi-square test was used to compare the proportions between the two periods.

The proportion of women who were tested for syphilis increased significantly from 52.8% (6890/13,059) in the risk-based testing period to 67.4% (9725/14,422) (p < 0.0001) in the opt-out period. The annual proportion of women who were tested for syphilis increased significantly from 51.9% (2163/4164) in 2015 to 68.3% (2306/3377) in 2020 (p_trend_<0.001) (Table 2). Syphilis positivity was similar in the risk-based testing period (0.48%, 33/6890) and opt-out period (0.71%, 69/9725) (p = 0.061) (p_trend_ = 0.003). Of the 102 syphilis cases, 100 (98%) cases were staged based on sufficient clinical evidence and the results of the blood test (13 primary, 14 secondary, 19 early latent and 54 late latent, Table 3). The proportion of cases that were late latent increased significantly from 36.4% (12/33) in the risk-based testing period to 60.9% (42/69) in the opt-out period (p = 0.033); while the proportion of primary (p = 0.342), secondary (p = 0.766) and early latent (p = 0.055) syphilis did not differ between the two periods.

**Table 2:** Syphilis testing and positivity among women tested for chlamydia before and after initiation of opt-out testing.

	Risk-based testing period			Opt-out testing period			Overall	p_trend_
Year	2015	2016	2017	2018	2019	2020	Total	
Number of women, N	4164	4535	4360	5128	5917	3377	27,481	–
Women tested for syphilis, n (%)	2163 (51.9%)	2259 (49.8%)	2468 (56.6%)	3441 (67.1%)	3978 (67.2%)	2306 (68.3%)	16,615 (60.5%)	<0.0001
Women who tested positive for syphilis, n (%)	5 (0.2%)	8 (0.4%)	20 (0.8%)	22 (0.6%)	25 (0.6%)	22 (1.0%)	102 (0.6%)	0.003

**Table 3:** The number and proportion of syphilis cases by stage in the risk-based testing and opt-out testing periods.

Syphilis stage	Risk-based testing period (N = 33)	Opt-out testing period (N = 69)	Total (N = 102)
Primary	6 (18.2%)	7 (10.1%)	13 (12.7%)
Secondary	5 (15.2%)	9 (13.0%)	14 (13.7%)
Early latent	10 (30.3%)	9 (13.0%)	19 (18.6%)
Late latent	12 (36.4%)	42 (60.9%)	54 (52.9%)
Unknown	0 (0%)	2 (2.9%)	2 (2.0%)

Table 4 shows the proportion of women who were tested for syphilis in the opt-out period by different characterises. Overall, the syphilis testing uptake was similar across the groups with different characteristics. However, there was a pattern that women with unknown and missing data on certain characteristics had a slightly lower syphilis testing uptake compared to those with completed data (e.g. those with missing data on pregnancy status, injecting drug use history, condom use and sex overseas).

**Table 4:** Syphilis testing uptake by characteristics among 14,422 women in the opt-out period.

Characteristics	n/N (%)
Age	
16–24	3645/5928 (61.5%)
25–34	5129/7085 (72.4%)
35–44	678/978 (69.3%)
≥45	273/431 (63.3%)
Aboriginal and/or Torres Strait Islanders	
No	7695/11,641 (66.1%)
Yes	99/171 (57.9%)
Unknown/missing	1931/2610 (74.0%)
Country of birth	
Australia	2276/3843 (59.2%)
Overseas	6676/9301 (71.8%)
Unknown/missing	773/1278 (60.5%)
Tested for urogenital chlamydia	
No	210/1168 (18.0%)
Yes	9515/13,254 (71.8%)
Living with HIV	
No	9695/14,391 (67.4%)
Yes	30/31 (96.8%)
Reported ever injecting drugs	
Never injected	9108/13,314 (68.4%)
Ever injected	108/153 (70.6%)
Unknown/missing	509/955 (55.3%)
Pregnancy status	
Not Pregnant	8308/12,223 (68.0%)
Pregnant	68/90 (75.6%)
Unsure	688/945 (72.8%)
Unknown/missing	661/1164 (56.8%)
Gender of sexual partners in the last 12 months	
Male only	8076/11,879 (68.0%)
Female only	119/198 (60.1%)
Both male and female	841/1112 (75.6%)
Unknown/missing	689/1233 (55.9%)
Condomless sex with any male partner in the last 12 months	
No	1140/1605 (68.4%)
Yes	7630/11,155 (71.0%)
Unknown/missing	955/1662 (57.5%)
Number of male partners in the last 12 months	
0	930/1593 (58.4%)
1–3	4258/6583 (64.7%)
≥4	4537/6246 (72.6%)
Had regular male partners	
No	4952/6986 (70.9%)
Yes	3613/5505 (65.6%)
Unknown/missing	1160/1931 (60.1%)
Had casual male partners in the last 12 months	
No	1066/1888 (56.5%)
Yes	7777/11,194 (69.5%)
Unknown/missing	882/1340 (65.8%)
Sex overseas in the last 12 months	
No	4354/6791 (64.1%)
Yes	4544/6246 (72.8%)
Unknown/missing	827/1385 (59.7%)

## Discussion

There have been public health concerns about the re-emergence of congenital syphilis and the rises in syphilis cases among women in Australia since the late 2010s. Current guidelines do not recommend screening asymptomatic heterosexual women outside of antenatal testing during pregnancy; therefore, strategies targeting heterosexuals are required for syphilis control.^20^ To our best knowledge, this is the first study to compare syphilis testing uptakes using risk-based testing and opt-out testing strategy exclusively among women attending a sexual health clinic setting in Australia. We found that there was a significant increase in the proportion of women tested for syphilis after changing from a risk-based testing to an opt-out testing strategy but there was no change in syphilis positivity between the two periods. This suggests that syphilis was detected in lower-risk women who would not have been tested during the risk-based testing period. Consistent with this was the observation that the opt-out testing strategy picked up significantly more asymptomatic late latent syphilis cases. These data support the opt-out testing strategy.

These results are comparable to existing literature that examined opt-out strategies in different at-risk populations. An Australian study from 46 sexual health clinics reported that syphilis testing among MSM living with HIV increased significantly from 27% in 2007 to 73% in 2014 (p_trend_<0.0001), after the recommendation of opt-out serological screening for syphilis on HIV viral load testing among PLHIV in 2009.^16^^,^^21^ The authors also revealed that the opt-out syphilis testing approach yielded an increase in the detection of asymptomatic syphilis infections and also a relative reduction in secondary syphilis.^16^ Additionally, Guy et al. reported that integrating the opt-out syphilis testing strategy as part of routine HIV management checks has significantly increased the syphilis testing rate (from 15% to 82%) among MSM living with HIV attending urban metropolitan clinics across Melbourne and Sydney.^17^ The opt-out strategy not only increases the syphilis testing rate, but also increases the annual syphilis testing frequency (i.e. 48% of men had ≥3 syphilis tests per year using the opt-out testing strategies compared to 39% of men had ≥3 syphilis tests per year using the opt-in testing strategies).^17^ A US study implemented a universal opt-out syphilis screening program among all patients attending an urban tertiary care hospital emergency department in Chicago.^22^ The authors found that women accounted for 33% of the syphilis cases, which was more than two times the national rate of 14% of cases in the US, suggesting some syphilis cases might have been missed from the traditional risk-based screening strategy. The authors further concluded a universal, opt-out screening for syphilis in high-prevalence populations should be considered for all adults.

It is important to note that a large proportion of syphilis cases in our study were asymptomatic. This is important because women with asymptomatic infection may not be aware of the infection without screening, and untreated syphilis may not only progress to the tertiary stage, but also can cause congenital syphilis via vertical transmission. Past studies on MSM have shown that an opt-out syphilis testing strategy has increased the detection of early latent syphilis^16^; however, we observed a greater detection of late latent syphilis cases rather than early latent syphilis cases, and this is likely because women are less frequent screened for syphilis compared to MSM and therefore have not had a syphilis test in the last 2 years. Our study illustrates that traditional identifiers of risk such as having sex overseas and pregnancy were associated with being tested for syphilis, which is consistent with the current screening recommendation.^12^

During the opt-out testing period, we found that syphilis testing uptake was similar among different groups. However, caution is needed in interpreting results as the sample size and therefore statistical power to detect differences between different groups is limited. Furthermore, we also found that there is a pattern of low syphilis testing uptake among women who declined to report certain practices (e.g. injecting drug use history, condom use and sex overseas). Past studies have shown that STI clinic attendees declined to report their sexual practices are tend to have higher rate of HIV/STI detection and therefore would be an important group to increase screening in.^18^

There were several limitations in this study. First, this study was conducted in an urban sexual health clinic and therefore, our findings may not be generalisable to other settings. A previous study has shown that 47% of syphilis cases in women in Victoria were diagnosed through general practice.^23^ General Practice (GP) clinics usually use the risk-based syphilis testing strategy to offer syphilis testing to women who are at risk. Given the large proportion of syphilis diagnoses made through GP, it is likely we were unable to ascertain if clients had previously presented to a health practitioner outside MSHC with symptoms or concerns. In such a scenario, clients may have opted out of syphilis testing because their perceived risk of exposure had not changed. This may be reflected in the non-significant increase in syphilis positivity. Second, some clients may have been dissuaded by the blood test due to the associated risks of blood taking. Third, we were unable to calculate the annual syphilis testing frequency because most women only attended the clinic once and annual syphilis testing is not currently recommended for all women. Third, the comparison of the proportion of different syphilis stages between the risk-based testing and opt-out periods must be interpreted with caution due to the small number of syphilis cases in both periods. Fourth, this was a pre-post observational study and therefore we could not rule out any time effect that might have caused an increase in syphilis testing in the opt-out period. Additionally, the opt-out period included 1 year at the beginning of the COVID-19 pandemic. Several studies have reported individuals had reduced their sexual practices and delayed their HIV/STI screening during the COVID-19 pandemic.^24^^,^^25^ In our study, there was a reduction in the total number of women attending our service in 2020 but this did not affect the proportion of women who were tested for syphilis. Future research should aim to further categorise participants’ refusal of syphilis testing in the opt-out period. This may provide valuable information about patient education and awareness of syphilis, and further understanding of how and where women present with syphilis.

In summary, our study reveals that an opt-out testing strategy is beneficial in increasing syphilis screening in women and importantly this increased screening resulted in an increase in the detection of cases. The increase in the detection of cases was particularly seen in asymptomatic late latent syphilis cases which is consistent with a beneficial effect of increased screening. Heterosexual women are typically not considered at risk for syphilis and hence the risk-based testing strategy is inadequate to detect these asymptomatic cases and prevent congenital syphilis not detected through antenatal care. On the basis of our findings, we suggest that the opt-out syphilis testing strategy is beneficial in sexual health services.

## Contributors

PG performed the data analysis and wrote the first draft of the manuscript. EPFC conceived and designed the study, and CKF provided input. EPFC provided statistical advice. EPFC has accessed and verified the data, and was responsible for the decision to submit the manuscript. All authors provided data interpretation, revised the manuscript for intellectual content, and approved the final version of the manuscript. The corresponding author had full access to all the data in the study and had final responsibility for the decision to submit for publication.

## Data sharing statement

The data that support this study are included in the article. Further inquiries can be directed to the corresponding author and the availability of the data will be subjected to the permission of the Alfred Hospital Ethics Committee.

## Declaration of interests

ELP has received an early-career research grant from Jack Brockhoff Foundation outside the submitted work. CSB has received the Australian Research Council (ARC) Industrial Research Transformation Hub Grant outside the submitted work, she has also received honorarium from Abbott outside the submitted work. EPFC has received investigator-initiated grants from MSD outside the submitted work, he has also received honorarium from MSD and CSL Seqirus outside the submitted work. All other authors have no conflicts of interest to declare.
